# Developmental outcomes of preterm infants with bronchopulmonary dysplasia-associated pulmonary hypertension at 18–24 months of corrected age

**DOI:** 10.1186/s12887-019-1400-3

**Published:** 2019-01-17

**Authors:** Eui Kyung Choi, Seung Han Shin, Ee-Kyung Kim, Han-Suk Kim

**Affiliations:** 1 Department of Pediatrics, Korea University Ansan Hospital, Korea University College of Medicine, 123, Jeokgeum-ro, Danwon-gu, Ansan-si, Gyeonggi-do Republic of Korea; 2 Department of Pediatrics, Seoul National University Children’s Hospital, Seoul University College of Medicine, 101, Daehak-ro, Jongno-gu, Seoul Republic of Korea

**Keywords:** Preterm infant, Neurodevelopmental outcome, Bronchopulmonary dysplasia, Pulmonary hypertension, Bayley scales

## Abstract

**Background:**

Owing to advances in the critical care of premature infants with bronchopulmonary dysplasia (BPD), BPD-associated pulmonary hypertension (PH) is becoming a growing concern. However, only few investigations were available on neurodevelopmental outcomes in preterm infants with PH. Therefore, this study aimed to identify the impact of PH on growth and neurodevelopment at 18–24 months of corrected age (CA).

**Methods:**

We retrospectively analyzed the medical records of 394 infants (aged < 28 weeks of gestation) admitted to the neonatal intensive care unit between 2005 and 2014. Among the surviving infants, 123 returned for follow-up evaluations including the *Bayley Scales of Infant and Toddler Development,* third Edition (Bayley-III) screening tests and growth assessment at 18–24 months of CA. Among the 81 infants with moderate or severe BPD, 20 met the criteria for PH. Baseline characteristics and outcomes were compared in infants who developed BPD-associated PH (PH group, *n* = 20) and moderate or severe BPD infants who did not develop PH (non-PH group, *n* = 61).

**Results:**

Compared to the non-PH group, the PH group showed significantly lower cognitive (85 vs. 95, *p* = 0.004), language (81 vs. 89, *p* = 0.040), and motor (88 vs. 94, *p* = 0.010) scores of the Bayley-III at 18–24 months of CA. Cognitive delay was found in 45.0% (9/20) of PH infants. In addition, z-scores of weight (− 1.4 ± 1.3 vs. -0.6 ± 1.1%, *p* = 0.011) and HC (− 1.2 ± 1.8 vs. 0.53 ± 1.0%, *p* = 0.035) were significantly lower in the BPD with PH group. With the subgroup analysis in infants with severe BPD only, the cognitive score was consistently lower and poorer and weight gain after discharge was identified in infants with PH and severe BPD.

**Conclusion:**

PH was a worsening factor of non-optimal growth and poor neurodevelopmental outcome in preterm infants with BPD at 18–24 months of CA. Our findings suggest the importance of close developmental follow-up and recognition of that risk to help optimize the outcome of preterm infants with PH.

## Background

Improved critical care management of premature infants has led to an increased survival rate of patients with bronchopulmonary dysplasia (BPD), which is the leading cause of late respiratory morbidity in preterm infants [1]. Early injury of the developing lung impairs angiogenesis and alveolarization, which in turn contributes to the development of BPD and BPD-associated pulmonary hypertension (PH). PH is a significant cardiovascular complication in infants with BPD and is associated with increased morbidity and mortality, such as longer hospitalization and oxygen therapy [2–4]. Recent efforts to identify PH in infants with BPD using echocardiography have shown that this modality may provide an opportunity for implementation of preventive or treatment strategies to improve long-term outcomes [5]. Despite the increased concern for PH, long-term outcomes in infants with BPD-associated PH remain unclear. Recently, several studies have reported cardiovascular outcomes of PH in preterm infants; however, no large longitudinal study that reported long-term pulmonary or neurodevelopmental outcomes in these infants is currently available [6, 7].

Since the association between BPD and poor neurodevelopment has already been established, children with BPD have higher rates of cognitive, educational, and behavioral impairments [8, 9]. However, with diverse interactions known to occur between prenatal and postnatal factors, the influence of PH in BPD on growth and neurodevelopmental outcomes has not been well established [4, 10–12]. Recently, Nakanishi et al. suggested in a current retrospective study that BPD with PH is a possible independent risk factor for neurodevelopmental impairment at 3 years of age [13].

The aim of the present study was to determine somatic growth and developmental outcomes in infants with BPD-associated PH at 18–24 months of corrected age (CA), highlighting their differences from infants with BPD, but without PH.

## Methods

### Study population

We retrospectively reviewed medical records of 394 preterm infants who were born at < 28 weeks’ gestational age and were admitted to the neonatal intensive care unit of Seoul National University Children’s Hospital between January 2005 and December 2014. Infants with major congenital anomalies, chromosomal abnormalities, and incomplete medical records were excluded. Additional exclusion criteria were missing neurodevelopmental assessment and growth data at 18–24 months of CA and severe neurological injury.

### Data collection and definitions

The following clinical data of infants were collected: birth weight, gestational age, small for gestational age (SGA; birth weight < 10th percentile for age according to Fenton growth charts), prenatal steroids (administration of any dose of corticosteroids during the concurrent pregnancy), histological chorioamnionitis (histopathological evidence of the presence of acute inflammatory changes in membrane roll and placental chorionic plate), and oligohydramnios (amniotic fluid index < 5 cm detected by ultrasonography performed just before delivery). The comorbidities of preterm infants were also assessed, such as respiratory distress syndrome (RDS; the presence of respiratory distress, increased oxygen requirement, and radiological findings consistent with RDS), patent ductus arteriosus (PDA) and its treatment, intraventricular hemorrhage (IVH; grading according to Papile’s classification [14]), culture-proven sepsis (determined at least in a single positive blood culture, and clinical signs of infection), retinopathy of prematurity, and necrotizing enterocolitis (NEC; according to modified Bell’s criteria [15]). BPD was defined as per the National Institute of Health consensus definition and graded as mild, moderate, or severe, according to the fraction of inspired oxygen (FiO_2_) or positive pressure ventilation (PPV) [16]. Mild BPD was defined as breathing room air, moderate BPD was defined as FiO2 of < 0.30, and severe BPD was defined as FiO2 of ≥0.30 or PPV at 36 weeks’ post-menstrual age. Patients with PDA were treated with cyclooxygenase inhibitors or surgical ligation. The following factors were used to estimate short-term respiratory prognosis: postnatal steroid use, duration of mechanical ventilation, home oxygen therapy, and total amount of oxygen supplementation until 36 weeks’ postmenstrual age calculated as supplemented extra oxygen concentration [%] (fraction of inspired oxygen – 21) × duration [h] [17]. The episodes of hypoxemia (defined as single or consecutive values of SpO_2_, < 80%) and bradycardia (pulse rate, < 80 /min) not recovering spontaneously were reviewed until 36 weeks’ post-menstrual age or discharge in infants with severe BPD.

### Pulmonary hypertension

Monthly echocardiographic examinations of preterm infants with BPD were conducted to screen for PH. Serial echocardiographic data of all preterm infants with BPD were reviewed, including two-dimensional, M-mode, and color-coded Doppler evaluation performed by a pediatric cardiologist at Seoul National University Children’s Hospital. Infants were diagnosed with PH if an echocardiogram performed at age older than 2 months demonstrated elevated pulmonary artery pressure based on the presence of at least one of the following criteria: 1) the velocity of tricuspid valve regurgitation of ≥3 m/s in the absence of pulmonary stenosis or 2) flat or left-deviated interventricular septal configuration and right ventricular hypertrophy with chamber dilation [18].

### Growth and neurodevelopmental assessment

Preterm infants discharged home were evaluated at 18–24 months of CA by one neonatologist in the neonatology outpatient clinic. Assessments included composite scores on the *Bayley Scales of Infant and Toddler Development*, third edition (Bayley-III) [19], and growth parameters (body weight, head circumference [HC], and length). Cognitive, language, and motor delay was defined as a composite score of < 85 [one standard deviation (SD) below the mean of 100] on the Bayley-III. Growth data were presented as z-scores, because infants were assessed at different gestational ages at birth and approximately at 18–24 months of CA. Fenton preterm growth charts were used as reference values from 22 to 50 gestational weeks, and the World Health Organization (WHO) Anthro software (WHO, Geneva, Switzerland) was used from term age onward. According to the WHO growth definition, underweight was defined as a z-score of ≤2.0.

### Statistical analysis

Data analysis was performed using SPSS 20.0 for Windows (SPSS Inc., Chicago, IL, USA). Continuous variables were analyzed using either the t-test or the Mann-Whitney U-test for normal or skewed distributions. Proportions were tested using chi-squared test and Fisher’s exact test. *P*-values of < 0.05 were considered statistically significant. Data were presented as mean ± SD, median and range, or rate.

## Results

**Fig. 1:**
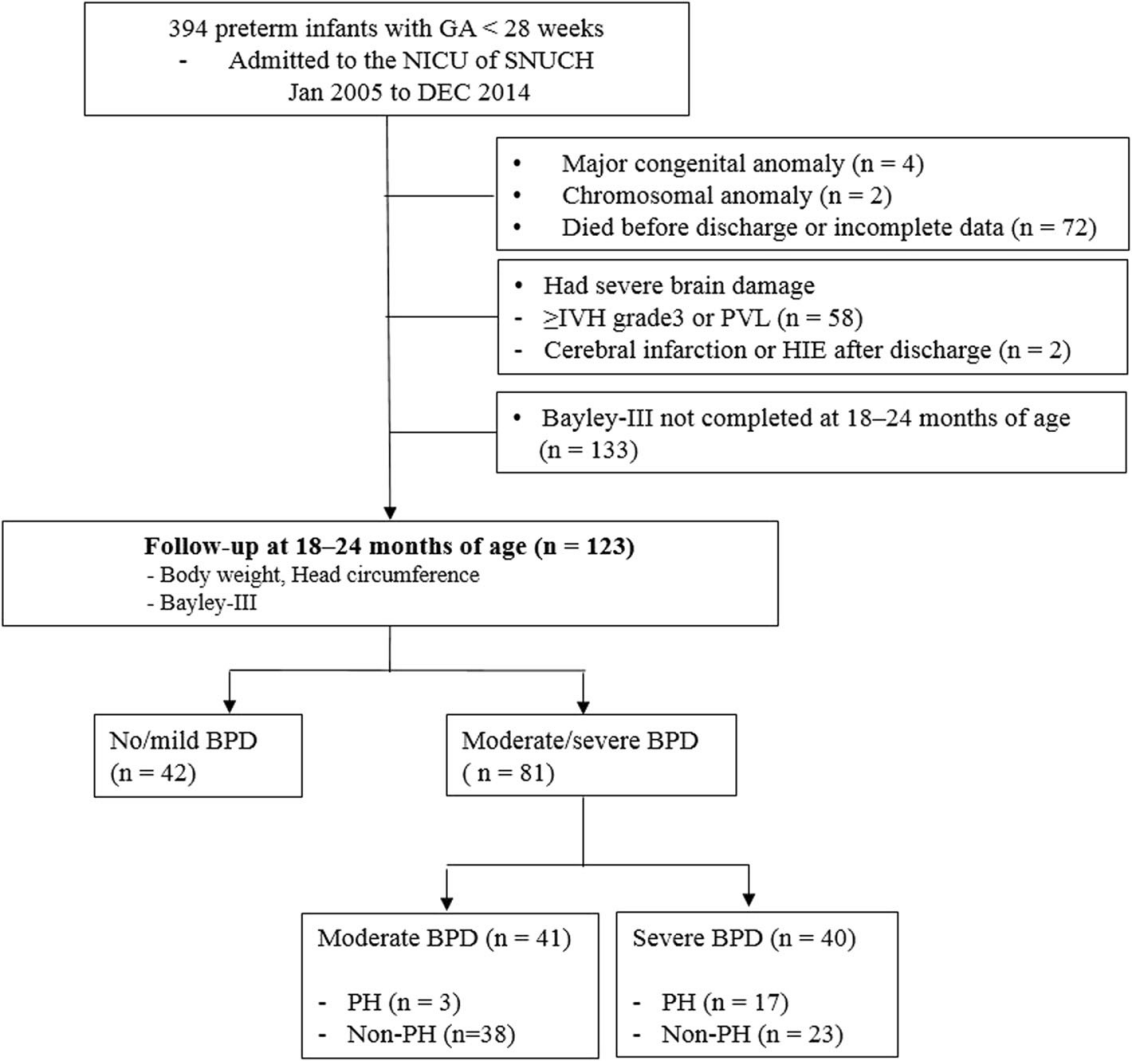
Flow chart of the study population

Of the 394 preterm infants born before 28 weeks of gestation, 6 were excluded due to major congenital malformations and chromosomal anomalies and 72 died before discharge or had incomplete data (Fig. 1). A total of 60 infants with grade III or IV IVH, periventricular leukomalacia, cerebral infarction, or hypoxic ischemic encephalomalacia diagnosed by cranial ultrasound or brain MRI were excluded to control for neurologic complications. Among the surviving infants, 123/256 (48.0%) returned for follow-up evaluations at 18–24 months of CA. In 123 preterm infants, 81 (66%) were classified with severe (40 of 81 infants) or moderate (41 of 81 infants) BPD. PH was diagnosed in 43% (17/40) of infants with severe BPD, 7% (3/41) of infants with moderate BPD, and 0% (0/42) of infants with no or mild BPD. Among the 81 infants with moderate or severe BPD, 20 (25%) met the criteria for PH. A flow chart showing the study design is presented in Fig. 1.

Clinical characteristics and short-term morbidities of infants with and without PH are presented in Table 1. No difference was observed in gestational age (*p* = 0.126), antenatal steroid exposure (*p* = 0.601), or post-natal steroid exposure (*p* = 0.128) for infants affected by BPD with PH compared to those affected by BPD without PH. Severe BPD was more prevalent in the PH group than in the non-PH group (85% vs. 15%, *p* <  0.001). Fifty-five percent of infants with PH had a history of culture proven sepsis, which was significantly higher than the number of infants without PH (*p* = 0.011). The median duration of hospitalization was longer in the PH group when compared to the non-PH group (*p* = 0.044). Table 2 shows the growth and developmental outcomes of infants with and without PH and BPD at 18–24 months of CA. Mean z-scores of weight (− 1.4 ± 1.3 vs. -0.6 ± 1.1%, p = 0.011) and HC (− 1.2 ± 1.8 vs. 0.53 ± 1.0%, *p* = 0.035) were significantly lower in the BPD with PH group than in the non-PH. In addition, the BPD with PH group showed significantly lower scores than the non-PH group in the cognitive (85 vs. 95, respectively, *p* = 0.004), language (81 vs. 89, *p* = 0.040), and motor (88 vs. 94, *p* = 0.010) areas of the Bayley-III. The prevalence of cognitive delay (45.0% vs. 9.8%, *p* <  0.001) was significantly higher in the BPD with PH group than those in the non-PH group.

**Table 1:** Clinical characteristics of subjects with and without pulmonary hypertension in infants with moderate/severe BPD

	PH	Non-PH	*P*-values
	(*n* = 20)	(*n* = 61)	
Gestational age, weeks	25.3 ± 1.4	25.8 ± 1.1	0.126
Birth weight, g	710.1 ± 183.6	758.6 ± 159.1	0.257
Birth weight < 10th percentile for age, n (%)	5 (25.0)	9 (14.8)	0.317
Multiple birth, n (%)	3 (15.0)	35 (57.4)	0.002
Cesarean section, n (%)	13 (65.0)	35 (57.4)	0.608
Perinatal steroids administration, n (%)	15 (75.0)	42 (68.9)	0.601
Chorioamnionitis, n (%)	12 (60.0)	25 (41.0)	0.138
Preeclampsia, n (%)	3 (15.0)	6 (9.8)	0.750
Oligohydramnios, n (%)	4 (20.0)	3 (5.1)	0.059
RDS, n (%)	16 (80.0)	45 (73.8)	0.767
Treated PDA, n (%)	16 (80.0)	47 (77.0)	1.000
BPD, n (%)			< 0.001
Moderate	3 (15.0)	38 (62.3)	
Severe	17 (85.0)	23 (37.7)	
Culture proven sepsis, n (%)	11 (55.0)	15 (24.6)	0.011
ROP operation, n (%)	7 (35.0)	34 (55.7)	0.128
NEC operation, n (%)	6 (30.0)	6 (9.8)	0.063
BPD steroid, n (%)	5 (25.0)	6 (9.8)	0.128
Length of stay, days	111 (82–268)	103 (71–163)	0.044

Data are presented as mean ± SD, median and range, or rate

*PH* pulmonary hypertension, *RDS* respiratory distress syndrome, *PDA* patent ductus arteriosus, *BPD* bronchopulmonary dysplasia, *ROP* retinopathy of prematurity, *NEC* necrotizing enterocolitis

**Table 2:** Growth and developmental outcomes of infants with or without pulmonary hypertension (PH) and moderate/severe bronchopulmonary dysplasia (BPD)

	PH	Non-PH	*P*-values
	(*n* = 20)	(*n* = 61)	
Growth at 18–24 months			
Body weight, kg	9.5 ± 1.5	10.2 ± 1.3	0.051
z-score	−1.4 ± 1.3	−0.6 ± 1.1	0.011
Head circumference, cm	44.9 ± 1.9	46.4 ± 1.5	0.001
z-score	−1.2 ± 1.8	−0.53 ± 1.0	0.035
Bayley-III at 18–24 months			
Cognitive score	85 (65–105)	95 (55–125)	0.004
Score < 85	9 (45.0)	6 (9.8)	< 0.001
Language score	81 (47–100)	89 (53–118)	0.040
Score < 85	10 (50.0)	20 (32.8)	0.167
Motor score	88 (52–107)	94 (46–115)	0.010
Score < 85	8 (40.0)	12 (20.3)	0.081

Table 3 shows the clinical characteristics according to the presence or absence of PH only in infants with severe BPD, which allowed to clarify the effects of PH. Among the infants with severe BPD, the clinical characteristics were statistically and significantly different between the infants with PH and those without PH in one variable: multiple births (17.6% vs. 60.9%, *p* = 0.010). The duration of mechanical ventilation (*p* = 0.090), the rate of infants who received dexamethasone rescue therapy (*p* = 1.000), the total amount of oxygen supplementation until 36 weeks’ postmenstrual age (*p* = 0.434), and the episodes of hypoxemia or bradycardia (*p* = 0.254 and *p* = 0.734, respectively) were not significantly different in the two groups. Table 4 shows the growth and developmental outcomes of infants with and without PH and severe BPD. At hospital discharge, no difference was found between the two groups according to the z-scores of the body weight and HC. However, at 18–24 months of CA, the mean z-score of body weight was significantly lower in infants with PH than in those without (− 1.7 ± 1.2 vs. -0.7 ± 1.3, *p* = 0.016). Compared to infants without PH, those with PH had lower cognitive scores (85 vs. 95, *p* = 0.048) only in Bayley-III.

**Table 3:** Clinical characteristics according to the presence or absence of PH in severe BPD

	Severe BPD		
	PH	Non-PH	*P*-values
	(*n* = 17)	(*n* = 23)	
Gestational age, weeks	25.3 ± 1.4	25.5 ± 1.0	0.470
Birth weight, g	709.5 ± 195.5	744.0 ± 158.6	0.543
Birth weight < 10th percentile for age, n (%)	5 (29.4)	4 (17.4)	0.456
Multiple birth, n (%)	3 (17.6)	14 (60.9)	0.010
Cesarean section, n (%)	12 (70.6)	13 (56.5)	0.512
Perinatal steroids administration, n (%)	13 (76.5)	17 (77.3)	1.000
Chorioamnionitis, n (%)	9 (52.9)	11 (47.8)	0.749
Preeclampsia, n (%)	3 (18.8)	2 (8.7)	0.631
Oligohydramnios, n (%)	4 (23.5)	2 (8.7)	0.373
RDS, n (%)	14 (82.4)	19 (82.6)	1.000
Treated PDA, n (%)	14 (82.4)	18 (78.3)	0.616
Culture proven sepsis, n (%)	9 (52.9)	8 (34.8)	0.251
ROP operation, n (%)	6 (35.3)	14 (60.9)	0.110
NEC operation, n (%)	5 (31.2)	3 (13.0)	0.235
Length of stay, days	115 (94–268)	113 (77–163)	0.448
Respiratory management			
Duration of CV or HFV, days	72 (0–199)	40 (6–148)	0.090
BPD steroid, n (%)	5 (29.4)	6 (26.1)	1.000
Dexamethasone cumulative dose (mg/kg)	1.80 (1.10–3.23)	1.10 (0.42–3.04)	0.416
Discharge on oxygen, n (%)	12 (70.6)	15 (65.2)	0.720
Total extra O_2_ supplementation^a^	25,199 (1428–71,095)	20,325 (11,417–46,362)	0.434
Episodes of hypoxia^b^	338 (172–1205)	258 (57–696)	0.254
Episodes of bradycardia^c^	40 (8–216)	56 (4–169)	0.734

Data are presented as mean ± SD, median and range, or rate

*PH* pulmonary hypertension, *BPD* bronchopulmonary dysplasia, *RDS* respiratory distress syndrome, *PDA* patent ductus arteriosus, *ROP* retinopathy of prematurity, *NEC* necrotizing enterocolitis, *CV* conventional ventilation, *HFV* high-frequency ventilation

^a^ Supplemented extra O_2_ concentration (%) (fraction of inspired O_2−_ 21%)

^b^ Single value or consecutive values of SpO2 < 80% until 36 weeks of postmenstrual age

^c^ Single value or consecutive values of pulse rate < 80 /min until 36 weeks of postmenstrual age

**Table 4:** Growth and developmental outcomes of infants with and without PH and with severe BPD

	PH	Non-PH	*P*-values
	(*n* = 17)	(*n* = 23)	
Growth at hospital discharge			
Body weight, kg	3.4 ± 1.0	2.9 ± 0.6	0.098
z-score	−2.2 ± 1.7	−2.1 ± 1.5	0.982
Head circumference, cm	33.7 ± 2.8	33.3 ± 1.8	0.663
z-score	−2.3 ± 1.5	− 1.8 ± 1.0	0.225
Growth at 18–24 months			
Body weight, kg	9.2 ± 1.4	10.1 ± 1.3	0.050
z-score	−1.7 ± 1.2	− 0.7 ± 1.3	0.016
Head circumference, cm	44.7 ± 2.0	46.0 ± 1.4	0.024
z-score	− 1.3 ± 1.9	− 0.7 ± 1.1	0.243
Bayley-III at 18–24 months			
Cognitive	85 (65–105)	95 (55–110)	0.048
Language	83 (47–100)	83 (53–115)	0.551
Motor	88 (52–97)	89 (46–110)	0.124

*PH* pulmonary hypertension, *BPD* bronchopulmonary dysplasia

## Discussion

This study found that the infant survivors with BPD-associated PH have significantly lower cognitive, language, and motor composite scores in the Bayley-III at 18–24 months of CA. In addition, the number of infants with cognitive delay was significantly higher in the BPD with PH group. Infants with BPD-associated PH had much lower body weights and HC than those with BPD only. Moreover, after adjusting for BPD severity, cognitive scores and body weight particularly remained lower in infants with PH and severe BPD. Recently, Nakanishi et al. [13] also reported that the developmental quotient (DQ) of < 70 in all areas using Kyoto Scale of Psychological Development (KSPD) was more prevalent and the body weight was lower than that in the non-PH group in 3-year-old infants with BPD-associated PH. However, no significant differences were detected in postural-motor, cognitive-adaptive, or language-social domain scores. In the present study, we found that not only a prevalence of cognitive score of < 85 was significantly higher but also the means of scores in cognitive, language, and motor areas were significantly lower in PH with moderate/severe BPD group, according to the Bayley-III scales, a global standard assessment method for preterm-born infants.

The severity of BPD has been recognized to be associated with poor neurodevelopmental outcomes. The mechanism may include multifactorial pathophysiology such as chronic, intermittent hypoxia associated with prolonged oxygen dependence leading to hypoxic-ischemic cerebral injury [20]. PH may exacerbate these complications because of hemodynamic instability and severe hypoxemia. In the present study, despite the fact that infants with BPD and PH had lower cognitive scores compared to those without PH, respiratory management, such as total extra oxygen supplementation, intermittent hypoxemia, and bradycardia events, was not different between the two groups. Although one study by Lodha et al. [21] showing that BPD with chronic oxygen dependency does not predict adverse neurodevelopmental outcomes compared to BPD only may support our result, causative pathophysiology on poor neurological outcomes by PH should be identified in a further study because peripheral capillary oxygen saturation (SpO_2_) and heart rate provide limited information on organ perfusion and systemic blood flow [22]. The further bedside neuro-monitoring technique includes near-infrared spectroscopy or amplitude-integrated electroencephalography to detect events of decreased cerebral oxygenation or changes in cortical activity could help to inform the risk of brain injury. Early identification of preterm infants at risk of neurologic injury by respiratory events in infants with PH will enable to initiate the neuroprotective strategies.

We also found that the body weight and HC were lower at 18–24 months of CA in the PH compared to non-PH group. Prenatal and postnatal restricted growth can be a potential explanation of neurodevelopmental disability. Placental insufficiency as manifested by SGA has been recognized as an important risk factor for BPD and PH [12]. Although the fact that SGA increases the risk of adverse neurodevelopmental outcomes in premature infants remains controversial, some studies reported increased levels of cognitive and behavioral difficulties in infants with SGA [23]. Furthermore, the association between postnatal growth failure and poorer neurodevelopmental outcomes in preterm infants has been well established in several large cohort studies [24, 25]. In the present study, the prevalence of SGA was not significantly different between the two groups, but poorer weight gain after discharge was identified in infants with PH and severe BPD compared to those with severe BPD only. Several mechanisms of growth failure have been proposed in infants with BPD: increased caloric expenditure in the work of breathing, restricted fluids, diuretic and postnatal steroid therapy, and different comorbidities, such as sepsis [26]. PH can be a causative or worsening factor especially in increasing caloric expenditure, restricted fluid or diuretic therapy in infants with severe BPD. The possible mechanism that PH causes failure of growth should be elucidated in a future prospective study.

In the total population analysis, the incidence of moderate or severe BPD was significantly higher in the PH group than that in the non-PH group. Because the BPD severity per se is known as a major risk factor for adverse neurological outcomes, the results in clinical outcomes should be cautiously interpreted by clarifying the effects of PH alone [8, 9, 13]. Therefore, a subgroup analysis was conducted on infants with severe BPD only to avoid the influence of GA, body weight, and mostly BPD severity, which demonstrated several interesting findings. The incidence of sepsis was significantly high in the PH group in the total population analysis; however, our subgroup analysis according to BPD severity showed that the two groups exhibited no difference in clinical characteristics. Furthermore, the language and motor outcomes were significantly lower in the PH group in the total population analysis but such results were not observed in the subgroup analysis. These finding might reaffirm that BPD severity per se is a major risk factor and many factors correlated with BPD severity may intricately play a role on adverse neurodevelopmental outcomes. The most notable point we demonstrated in the subgroup analysis in this study is to suggest that PH might be an additional risk factor for cognitive impairment in infants with severe BPD. Although other factors such as sepsis and NEC were not found as significant additional risk factors, the role of these factors cannot be concluded because of the retrospective design, small sample size, and low follow-up rate at 18–24 months of CA in this study. Further prospective large-scale studies might clarify the intricate correlation of these factors on the adverse neurodevelopmental outcomes in infants with severe BPD and PH.

## Conclusion

In summary, preterm infants with PH have shown significantly low scores in cognitive, language, and motor areas in the Bayley-III at 18–24 months of CA. With subgroup analysis in infants with severe BPD only, the cognitive score remained low in infants with PH. Furthermore, growth restriction is more likely to persist after discharge in severe BPD infants with PH than in infants with severe BPD only. Therefore, a more tailored approach on post-discharge neurodevelopment and growth should be implemented for long-term follow-up of preterm infants with BPD-associated PH. Additional prospective and large studies are needed to confirm our results and to provide information that improves the long-term outcomes of preterm infants with PH.

## Abbreviations

Bayley-III: *Bayley Scales of Infant and Toddler Development*, third edition
BPD: Bronchopulmonary dysplasia
CA: Corrected age
IVH: Intraventricular hemorrhage
NEC: Necrotizing enterocolitis
PDA: Patent ductus arteriosus
PH: Pulmonary hypertension
RDS: Respiratory distress syndrome
SGA: Small for gestational age
